# Effects of Interpersonal Sensorimotor Synchronization on Dyadic Creativity: Gender Matters

**DOI:** 10.3389/fpsyg.2018.02604

**Published:** 2019-02-04

**Authors:** Andrea Gaggioli, Elisa Maria Falletta, Francesco Ferrise, Serena Graziosi, Alberto Gallace, Alessandro D’Ausilio, Pietro Cipresso, Giuseppe Riva, Alice Chirico

**Affiliations:** ^1^Applied Technology for Neuro-Psychology Laboratory, Istituto Auxologico Italiano, Milan, Italy; ^2^Department of Psychology, Università Cattolica del Sacro Cuore, Milan, Italy; ^3^Department of Mechanical Engineering, Politecnico di Milano, Milan, Italy; ^4^Department of Psychology and Milan Centre for Neuroscience, University of Milano-Bicocca, Milan, Italy; ^5^Center of Translational Neurophysiology, Istituto Italiano di Tecnologia (IIT), Center for Translational Neurophysiology of Speech and Communication (CTNSC), Universitá di Ferrara, Ferrara, Italy; ^6^Sezione di Fisiologia Umana, Dipartimento di Scienze Biomediche e Chirurgico Specialistiche, Universitá di Ferrara, Ferrara, Italy

**Keywords:** dyadic creativity, interpersonal coordination, interpersonal synchronization, joint action, gender, networked flow

## Abstract

Although it is noted that interpersonal sensorimotor coordination can influence several high-level socio-cognitive processes, its impact on creative collaboration is nearly unexplored. Here, we investigated the effects of a form of sensorimotor coordination, that is, sensorimotor synchronization, on a subsequent creative collaboration task. 60 pairs (*n* total = 120 participants) formed by previously unacquainted individuals performed a tower-building task either jointly or alone, followed by a dyadic creativity task. Tower building time in the joint condition was recorded through a sensorized platform and creativity performance was evaluated by two independent raters based on the quantity and quality of generated ideas. We controlled for gender composition and for the disposition to cooperate and to adopt a creative, analytical style. Results showed that male-male couples were more creative after the joint-action condition, whereas female-female and mixed-gender couples were more creative after the solo condition. Regression analyses of tower building time on creativity performance revealed that building time was a significant predictor of creativity dimensions in male-male and in mixed-gender couples but did not predict creative performance in female-female couples. Overall, these findings suggest that the manipulation of sensorimotor coordination can influence performance in a subsequent creative collaboration task, with the nature, and magnitude of this effect depending on the gender composition of the dyads. These results have potential implications for the design of sensorimotor-based strategies to enhance dyadic creative performance in several contexts, especially for the organizational settings.

## Introduction

Joint action can be defined as the ability to act together with others and it is key to several social action behaviors, such as group dancing, music ensemble performance, surgical operations, and team sports (Sebanz and Knoblich, 2009; Repp and Su, 2013). In these joint activities, sensorimotor coordination – here defined as a temporal synchronization of body movements between individuals involved in social interactions (Bernieri et al., 1988) – is a key component. Synchronization among people during social interactions is one of the primary forms of interpersonal coordination (Miles et al., 2017). In dynamical systems theory, behavioral synchronization is regarded as an emergent phenomenon that can be modeled as a system of two coupled oscillators (Schmidt et al., 1990). This perspective has been extended to highlight the critical role of mutual behavioral prediction and thus the continuous and flexible exchange of bodily signals, to enhance sensorimotor coordination (Pezzulo et al., 2018). That is, both bodies and minds are involved to achieve interpersonal synchronization (Sebanz et al., 2006; Repp and Su, 2013).

Crucially, research in the domain of social embodied cognition has theorized and shown that even low-level behavioral synchronization can positively modulate specific high-level social processes, such as cooperation (Semin and Cacioppo, 2008; Wiltermuth and Heath, 2009; Valdesolo et al., 2010) affiliation (Hove and Risen, 2009), altruism, and empathy (Valdesolo and DeSteno, 2011). According to this perspective, also creativity can be considered as an embodied high-order cognitive process, which is influenced also, and especially, by individuals’ body (Stanciu, 2015). Emerging theoretical models are increasingly examining the role of interpersonal coordination on creative collaboration and in particular on dyadic creativity. For example, the Networked Flow model developed by Gaggioli et al. (2013a,b,c, 2015, 2017), (Sawyer, 2003, 2007; Galimberti et al., 2015), highlights the importance of *social presence* (i.e., the feeling of being and acting “together”; Biocca and Harms, 2003) and *mutual engagement* (i.e., group flow; Sawyer, 2003, 2007) as factors facilitating interpersonal creativity. Similarly, Rouse (2018) theorized the role of a “psychological pairing” supported by a constant feedback loop (Harrison and Rouse, 2014) at the base of interpersonal intimate co-creation process in which ideas *flow* continuously between partners.

On the empirical level, the link between primary forms of interpersonal coordination – behavioral synchronization – and dyadic creativity has started to be investigated only recently. Won et al. (2014) used automatically detected measures of synchrony – which they defined as “the temporal linkage of the non-verbal behavior of two or more interacting individuals” to retrospectively predict performance in a creative collaborative task, in which dyads were invited to generate novel strategies to conserve resources. Results of this experiment showed a significant relationship between spontaneous coordination and creativity, indicating that higher behavioral synchronization was associated with a higher number of new and valid ideas produced by the couples. Interestingly, the authors highlighted that synchronous behavior may be linked to the concept of rapport, defined as “a state of mutual positivity and interest that arises through the convergence of non-verbal expressive behavior in an interaction” (Drolet and Morris, 2000, p. 27). In the same vein, Weinstein et al. (2010) showed that connectedness between partners (i.e., the extent to which partners feel close), their mutual engagement, the presence of responsive interaction and their level of wellbeing were all significant predictors of creative dyadic performance.

The presents study adds to the emerging literature on sensorimotor synchronization and interpersonal creativity by investigating the direct impact of *induced* behavioral interpersonal synchronization on a *subsequent* dyadic creativity task. Here, we conceived interpersonal creativity as the generation of novel, original, useful, and feasible products by means of some sort of collaborative process (Torrance, 1966; Amabile, 1996; Paulus et al., 2010) raging from the one arising from a brainstorming session in a group of businessman or designers (Rawlinson, 2017) or from a couple of individuals. Moreover, as concerns the creative outcome, we defined creativity also in terms of *fluency*, that is, the number of ideas produced to solve a problem, *flexibility*, i.e., number of different conceptual categories arising from a set of ideas, and *elaboration*, that is, the number of details associated to a single idea (Guilford, 1950; Torrance et al., 1989). We chose to work with couples as dyads provide the basic prototypical condition for studying interpersonal synchronization arising from a joint action task in a controlled setting. Moreover, although few studies on diversity and dyadic creativity exist (e.g., Cohen et al., 1960; Hoffman and Maier, 1961; Triandis et al., 1965; Torrance, 1970), it is still unclear how creativity takes place in dyads, especially in the organizational field (Rouse, 2018). Dyads may display different processes enabling collaborative creative work.

Based on previous theoretical models linking interpersonal coordination and creative collaboration (Gaggioli et al., 2013a,b,c; Rouse, 2018), we argued that the flow of sensorimotor information occurring between two interacting partners involved in a joint motor task (i.e., building a tower together) may facilitate co-generation of ideas, resulting in higher levels of dyadic creativity. In short, we expected that pairs who had been primed with a synchronization-conductive task would generate more and better ideas than pairs who had performed the same task alone. In testing this hypothesis, we also wanted to control for diverse set of factors that may influence dyadic cooperation, such as disposition to cooperate, cognitive style, perceived interpersonal attraction, and gender. Actually, increasing evidence shows that gender modulates patterns of synchronization in dyads both at behavioral and at neural level (e.g., Abney et al., 2015; Cheng et al., 2015, 2017; Cornejo et al., 2017; Fishburn et al., 2018). For instance, mixed pairs showed significant correlations between increased brain synchronization and high cooperative performance (Cheng et al., 2015). Furthermore, gender composition influenced dyadic performance differently (Strough and Diriwächter, 2000), although effects of gender diversity on dyadic performance have been discussed for a long time (e.g., Cohen et al., 1960; Hoffman and Maier, 1961; Triandis et al., 1965; Torrance, 1970). For instance, a study by Weinstein et al. (2010) showed that stimulating pairing in couples with an orientation task (i.e., autonomy vs. control vs. no-orientation) can enhance their subsequent creative performance in relation to gender-composition, although authors did not fully elucidate the role of gender. Accordingly, in the current study, we did not formulate specific hypotheses on the role of gender-diversity, but we adopted an explorative approach.

## Materials and Methods

### Participants

120 participants, 60 males (mean age = 23.32; *SD* = 1.91), and 60 females (mean age = 22.48; *SD* = 1.53) took part to the experiment. Participants were undergraduate students recruited through campus announcements at universities sited in Milan. Participants were assigned to couples randomly, after matching these criteria: (i) couple members were previously unacquainted individuals and could not talk each other before the beginning of the experiment; (ii) couple members had the same manual dominance (both right-handed or left-handed). Three types of couples were compared: male couples, female couples, and mixed-gender couples. The experimental protocol was approved by the Ethical Committee of the Università Cattolica del Sacro Cuore prior to data collection. Each participant provided written informed consent for study participation in accordance with the Helsinki Declaration. No reward was provided to participants.

### Measures

#### Pre-experiment Measures

Prior to experiment, to control for potentially intervenient effects of cooperation-related variables, we administered three questionnaires assessing: (i) disposition to cooperate, (ii) manual dominance, and (iii) cognitive style, as follows: Disposition to cooperate was assessed using the Cooperativeness scale of the Temperament and Character Inventory-Revised (TCI-R) (Cloninger et al., 1994; Fossati et al., 2007). Cooperativeness scale is composed by 36-items on a five-point Likert scale (1, absolutely false; 5, absolutely true). It includes five subscales (Social Acceptance; Empathy; Helpfulness; and Pure-hearted Conscience) and has a good internal consistency, with a total Cronbach Alpha of 0.85.

Handedness was assessed using a 12-items questionnaire (Briggs and Nebes, 1975). We controlled this dimension in order to match people with same handedness. The Cognitive Style Indicator (CoSI) (Cools and Van den Broeck, 2007) was used to control for the disposition to adopt a preferential cognitive style to solve problems. The CoSI distinguishes between three cognitive styles: (i) a knowing style, emphasizing logic, objectivity, and precision; (ii) a planning style, emphasizing structure, control, and routines; (iii) a creating style, emphasizing subjectivity, impulsivity, and openness to possibilities. These dimensions are measured through 18-items using five-point ratings (1 = it does not describe me at all; 5 = it describes me perfectly). The Cognitive Style Indicator has shown acceptable internal consistency, with Cronbach’s alpha coefficients ranging from 0.73 to 0.85.

#### Dyadic Creativity Task

Drawing on theorization of Amabile (1983) and Guilford (1950, 1959, 1967), and following the methodological guidelines provided by Hashemi Farzaneh et al. (2012), we designed an *ad hoc* task to measure dyadic creativity, which consisted in asking participants to generate as many ideas as possible about how to safely park a bicycle. Each dyadic brainstorming session was video-recorded for later analysis. Two independent raters were involved to assess ideas arising from pairs discussion using video-recorded materials. Raters did not already know each other and had different expertise: Rater 1 was a 25-years-old master graduated in Design & Engineering; Rater 2 was a 28-years-old medical doctor. Both raters were instructed to code the generated ideas using the following criteria:

(1) Quantity, which refers to the number of ideas generated;
(2) Appropriateness, which refers to the number of ideas considered appropriate to the task;
(3) Elaboration, which refers to the number of details associated to an idea.
(4) Feasibility, which refers to which extent an idea could be realized (1, absolutely impractical; 7, absolutely feasible).
(5) Usefulness, refers to the degree in which the solution is useful. Two independent judges were requested to state their opinion on a seven-point Likert scale (1, absolutely useless; 7, absolutely useful).
(6) Originality, refers to the extent to which every idea is rare. Two independent judges were requested to state their opinion on a seven-point Likert scale (1, absolutely common; 7, absolutely rare).
(7) Flexibility, refers to the extent to which the whole creative session was creative regarding the viewpoints adopted to approach the topic/problem assigned. Every semantic category to which ideas were attributable counted one score.

#### Post Experimental Measures

At the end of the experimental session, participants were required to report the extent to which they experienced interpersonal attraction by filling the Measurement of Interpersonal Attraction (McCroskey and McCain, 1974). This questionnaire includes 18-items on a five-point Likert scale (1, absolutely disagree; 5, absolutely agree) and is composed by three subscales: social attraction, physical attraction, and task attraction.

#### Procedure

**FIGURE 1:**
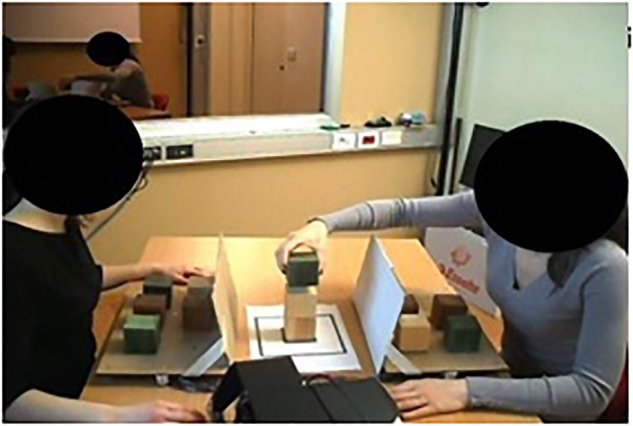
Couple involved in the tower-building task using the COLLEGO platform in the experimental condition (joint).

**FIGURE 2:**
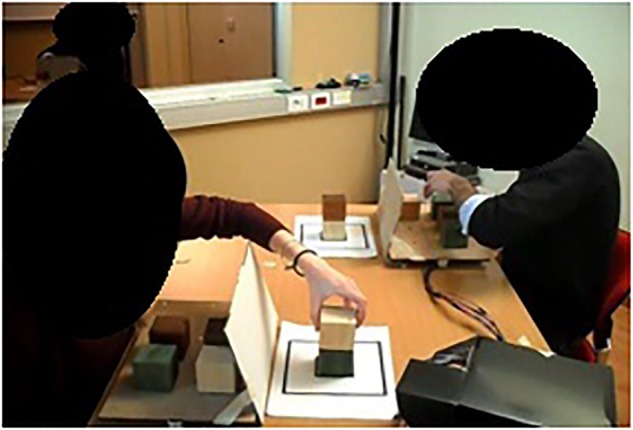
Couple involved in the tower-building task using the COLLEGO platform in the control condition (not-joint).

This study employed a between-subjects design. Pairs were randomly assigned to either the experimental condition or to a control one. Pairs assigned to the experimental condition were asked to build a tower together, as quick as possible, using a total of 12 colored cubes (thus, each participant used 6 cubes) (Figure 1). Couple members had to pay attention to not pick a cube before the partner had released the other one. Each trial, the experimenter assigned couple members their leader/follower role. The leader started choosing a color and the follower had to continue with the same color. In contrast, couples assigned to the control condition were required to build the tower individually. In both conditions, participants were instructed to build a total of 10 towers. The COLLEGO platform, a custom-made device developed to allow measuring participants’ moves and time during the execution of the task (D’Ausilio et al., 2015; Coco et al., 2016; Chirico et al., 2016). The experimental condition required participants to build the tower jointly. When a cube was picked/released, timestamp (ms), and position of the selected object was recorded. Participants in the control condition executed the same task facing each other, in the same room but independently (Figure 2). Thus, within this condition, each participant used only 6 of the 12 cubes available. At the end of the tower-building task, all pairs were involved in the creativity task (5 min length).

## Results

### Data Analyses

Analyses were done using IBM SPSS Statistics software (Version 21, release 21.0.0.0 64-bit edition). First, we conducted a normality test, testing kurtosis, and asymmetry for dyadic creativity measures (Quantity, Appropriateness, Elaboration, Feasibility, Usefulness, Originality, and Flexibility). We found that all variables followed a normal distribution.

### Creativity Measures

We estimated inter-rater reliability between the two raters for each creativity dimension, using Cronbach’s alpha. Internal consistency was from acceptable to high, with alpha coefficients ranging from 0.519 to 0.936 (Quantity: 0.936; Appropriateness: 0.913; Elaboration: 0.519; Feasibility: 0.659; Usefulness: 0.778; Originality: 0.577; Flexibility: 0.833). Thus, judges’ scores were aggregated to obtain a single value for each creativity dimension. Next, a Factorial ANOVA 2 (Condition: joint vs. non-joint) × 3 (Gender: Male-Male vs. Female-Female vs. Male-Female) was conducted on creativity dimension scores. Results showed that there was no main effect of Condition. A main effect for gender was found on three creativity dimensions: Quantity [*F*(2,54) = 3.183, *p* = 0.049; η^2^ = 0.083], Appropriateness [*F*(2,54) = 3.25, *p* = 0.046; η^2^ = 0.085], and Flexibility [*F*(2,54) = 8.525, *p* = 0.001; η^2^ = 0.192] in the joint condition. Specifically, *post hoc* comparisons using Bonferroni showed that M–M couples achieved significantly higher scores of Quantity (mean = 27.40; *SD* = 10.79) compared with M–F couples (mean = 21.25; *SD* = 7.30), and F–F couples (mean = 23.90; *SD* = 7.15). No significant difference was found between F–F and M–F couples in terms of Quantity. At the same time, M–M couples generated a significantly higher number of appropriate solutions (mean = 23.90; *SD* = 9.16) than M–F couples (mean = 18.70; *SD* = 6.08), and, than F–F couples (mean = 20.65; *SD* = 6.04). No significant difference emerged between F–F and M–F couples in terms of Appropriateness. Finally, ideas produced by M–M were characterized by significantly higher level of Flexibility (mean = 10.40; *SD* = 1.98) compared with those generated by F–F couples (mean = 8.10; *SD* = 2.25), and M–F couples (mean = 8.75; *SD* = 1.86) (see Table 1 for general descriptive statistics and Table 2 for *post hoc* analyses).

**Table 1:** Descriptive statistics of each creativity dimensions for each gender couple in both conditions: Mean and Standard Deviation.

Condition		Joint			Not-Joint		
			
Creativity dimension		MM	FF	MF	MM	FF	MF
Quantity	Mean	32.90	20.30	19.00	21.90	27.50	23.50
	SD	11.49	5.29	7.00	6.80	7.15	7.21
Appropriateness	Mean	28.60	18.20	16.10	19.20	23.10	21.30
	SD	10.29	4.42	6.14	4.73	6.64	5.03
Elaboration	Mean	2.81	3.18	3.44	2.98	2.51	2.89
	SD	0.631	1.18	1.05	0.52	0.34	0.52
Feasibility	Mean	9.87	10.50	10.04	10.47	10.59	10.38
	SD	0.92	2.01	1.09	1.18	0.94	0.89
Usefulness	Mean	9.39	9.21	9.06	9.33	8.90	9.13
	SD	0.49	0.60	1.08	1.02	0.621	0.82
Originality	Mean	7.27	7.05	7.53	7.64	7.06	7.30
	SD	0.86	1.61	1.00	1.05	0.96	0.76
Flexibility	Mean	11.30	7.10	7.70	9.50	9.10	9.80
	SD	2.11	2.18	1.25	1.43	1.91	1.81


*MM, male couples; FF, female couples; MF, mixed-gender couples.*

**Table 2:** *Post hoc* analysis of gender differences within each condition (joint vs. not-joint) concerning quantity, appropriateness, flexibility, duration.

	Joint			Not-Joint		
		
	MM vs. FF	MM vs. MF	FF vs. MF	MM vs. FF	MM vs. MF	FF vs. MF
Quantity	0.007	0.003	n.s	n.s	n.s	n.s
Appropriateness	0.012	0.002	n.s	n.s	n.s	n.s
Flexibility	0.00014	0.001	n.s	n.s	n.s	n.s
Duration	n.s	0.006	0.053	–	–	–


*Duration *post hoc* concerned only the joint condition. We reported only *p*-values of each significant comparison.*

**FIGURE 3:**
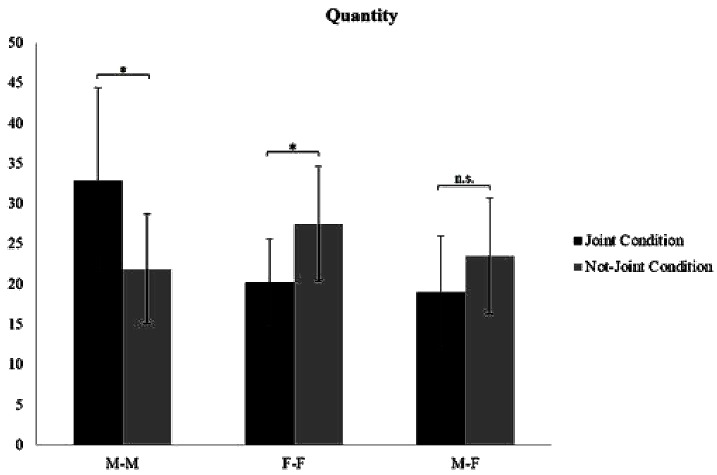
Interaction effect of Gender by Condition on creativity dimension of Quantity. M–M, comparison between male pairs in the joint vs. not-joint condition; F–F, comparison between female pairs in the joint vs. not-joint condition; M–F, comparison between mixed gender pairs in the joint vs. not-joint condition. ^∗^*p* < 0.05; ^∗∗^*p* < 0.01.

**FIGURE 4:**
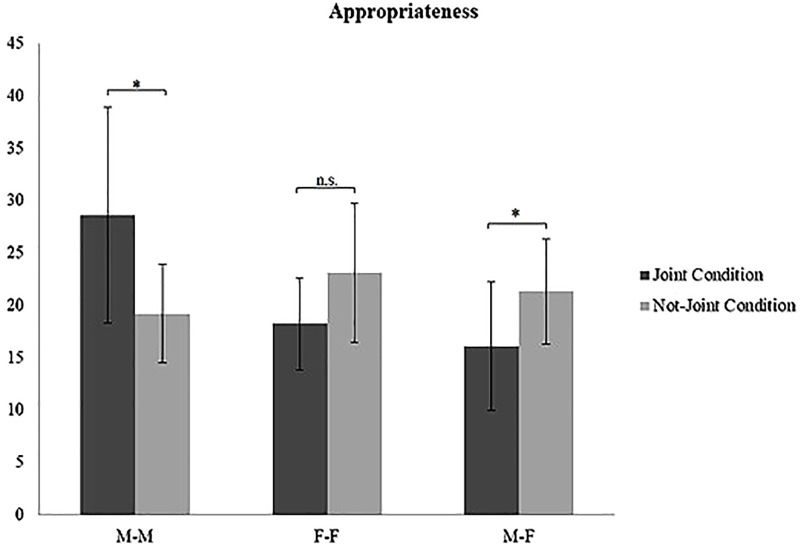
Interaction effect of Gender by Condition on the creativity dimension of Appropriateness. ^∗^*p* < 0.05.

**FIGURE 5:**
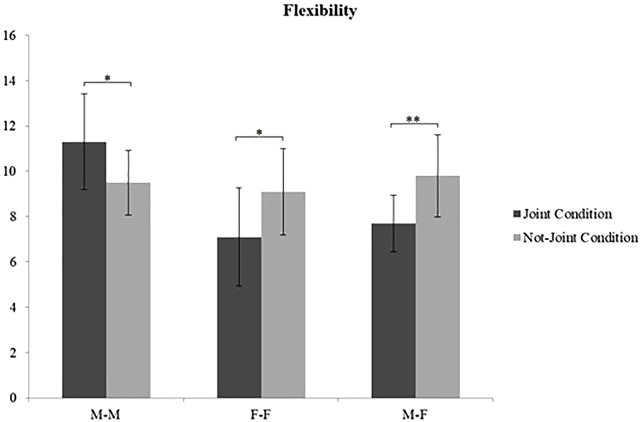
Interaction effect of Gender by Condition on creativity dimension of Flexibility. ^∗^*p* < 0.05; ^∗∗^*p* < 0.01.

A significant interaction effect was found between Condition and Gender for each creativity dimension: Quantity [*F*(2,54) = 8.066; *p* = 0.001; η^2^ = 0.211] (Figure 3), Appropriateness [*F*(2,54) = 8.196, *p* = 0.001; η^2^ = 0.213] (Figure 4), and Flexibility [*F*(2,54) = 7.494, *p* = 0.001; η^2^ = 0.169] (Figure 5). Specifically, M–M couples had higher Quantity scores in the joint condition (mean = 32.90; *SD* = 11.49) than in the non-joint one (mean = 21.90; *SD* = 6.80); the opposite result was found in F–F couples, which produced significantly more ideas in the non-joint condition (F–F couples mean = 27.50; *SD* = 7.15) than in the joint one (F–F couples mean = 20.30; *SD* = 5.29). M–F did not show differences regarding quantity in the not joint-condition (M–F couples mean = 23.50; *SD* = 7.21) compared to the joint condition (M–F couples mean = 19.00; *SD* = 7.00). M–M couples had higher Appropriateness scores in the joint condition (mean = 28.60; *SD* = 10.29) than in the non-joint one (mean = 19.20; *SD* = 4.73), whereas only M–F couples produced more appropriate ideas in the not-joint control condition (M–F couples mean = 21.30; *SD* = 5.03) than in the joint one (M–F couples mean = 16.10; *SD* = 6.14). M–M couples had higher Flexibility scores in the joint condition (mean = 11.30; *SD* = 2.11) than in the non-joint one (mean = 9.50; *SD* = 1.43). Again, F–F couples and M–F couples showed the opposite trend, adopting a significantly higher number of semantic categories in the non-joint condition (F–F couples mean = 9.10; *SD* = 1.91; M–F couples mean = 9.80; *SD* = 1.81) than in the joint one (F–F couples mean = 7.10; *SD* = 2.18; M–F couples mean = 7.70; *SD* = 1.25). The other creativity dimensions (i.e., Elaboration, Feasibility, Usefulness, and Originality) did not show any significant effect. Please see Table 3 for *post hoc* analyses on gender and condition (see Table 3 for *post hoc* referring to gender differences within each condition).

**Table 3:** *Post hoc* analysis of gender differences within each condition (joint vs. not-joint) concerning quantity, appropriateness, flexibility, duration.

	Males	Females	Mixed
	Joint vs. Not-Joint	Joint vs. Not-Joint	Joint vs. Not-Joint
Quantity	0.018	0.020	0.174
Appropriateness	0.017	0.068	0.05
Flexibility	0.039	0.043	0.007


*We reported only *p*-values of each significant comparison.*

### Tower-Building Time

A second set of analyses focused on determining differences in tower-building time within the joint-action condition. We computed the time each couple took to complete a single tower (trial) and calculated the average time across the 10 trials. A one-way ANOVA was conducted to determine if gender composition influenced average tower-building time. Results showed a significant effect of Gender on Time [*F*(2,27) = 6.320, *p* = 0.006]. *Post hoc* tests using Bonferroni correction revealed that same-gender couples (M–M: mean = 17668.78; *SD* = 7595.72; F–F: mean = 20529.71; *SD* = 5473.70) were significantly faster than mixed-gender couples (mean = 28528.73; *SD* = 7921.08), while no significant difference was found among M–M and F–F couples. To test the causal impact of tower-building time (“Time”) on creativity dimensions for each gender composition type (M–M; F–F; M–F), we carried out 21 Simple Linear Regression models considering “Time” as predictor and creativity dimensions as dependent variables. Results of Simple Linear Regression are reported in Table 1. The measure of Time resulted significant predictor of Appropriateness, Originality, and Flexibility in M–M couples, while it emerged as a significant predictor of Elaboration only in M–F couples. Please, see Table 4 for results of the Linear Regression models.

**Table 4:** Results of linear regression models describing the influence of tower-building time on creativity dimensions.

	Time											
	
	MM				FF				MF			
			
	Beta	*R*^2^	F	Sign.	Beta	*R*^2^	F	Sign	Beta	*R*^2^	F	Sign.
Quantity	-0.505	-0.595	4.380	0.07	–	–	–	–	–	–	–	–
Appropriateness	-0.625	0.391	5.127	0.053	–	–	–	–	–	–	–	–
Elaboration	–	–	–	–	–	–	–	–	-0.641	0.411	5.594	0.046
Originality	-0.728	0.530	9.02	0.017	–	–	–	–	–	–	–	–
Flexibility	-0.686	0.470	7.106	0.029	–	–	–	–	–	–	–	–


*n = 120. We reported only significant models.*

## Discussion

We evaluated the effects of prior interpersonal sensorimotor coordination (present or absent) on a subsequent creativity task, in dyads of varying gender composition. We hypothesized that the behavioral flow of information promoted by the joint-action task would be reflected into an enhanced flow of ideas between partners. This latter process dwells at the base of dyadic creativity. We also assumed that the creativity outcome would be shaped by pairs’ gender composition. Some authors (Miles et al., 2009; Gaggioli et al., 2013a,b, 2015; Chirico et al., 2016; Rouse, 2018) suggested that the experiential correlates of this process can be found in the emergence of a sense of rapport, or as a psychological pairing, or sense of mutual engagement and connectedness between members. Here, we focused on the direct link between behavioral and cognitive performance, in line with current perspectives of Embodied creativity (e.g., Won et al., 2014; Byrge and Tang, 2015; Noy et al., 2015; Stanciu, 2015). This research revealed the role of previous behavioral synchronization, as well as gender on dyadic creative performance. Results indicated: (i) a main effect of gender composition; (ii) no main effect of the sensorimotor coordination condition; and (iii) a significant interaction effect between prior task and gender composition. We will discuss these results in the same order. Gender composition affected key dimensions of creativity performance in the joint condition. Specifically, M–M couples generated more flexible solutions compared with those generated by F–F couples and M–F couples; moreover, M–M generated a higher number of ideas, which were also more appropriate and flexible, than female and mixed-gender couples. No differences in creativity performance were observed between F–F and mixed-gender dyads nor in the joint neither in the not-joint condition. At the sensorimotor level, same-gender couples outperformed compared to mixed-gender couples, but there was no significant difference between M–M and F–F in terms of behavioral performance.

Crucially, only half participants in the joint condition was primed with a behavioral synchronization task. Participants’ performance at the behavioral synchronization task in the joint condition was specular to participants’ performance after the creativity task. Specifically, gender-based pairs who performed the tower-building task together showed the same performance trend both during the behavioral task and after the subsequent dyadic creativity task: males outperformed compared to mixed couples in terms of tower-building time, as well as in terms of Quantity (i.e., M–M significantly outperformed compared to F–F and M–F), Appropriateness (i.e., M–M significantly outperformed compared to F–F and M–F), and Flexibility (i.e., M–M significantly outperformed compared to F–F and M–F) dimensions of creativity. Although no main effect of condition was found, a completely opposite pattern emerged for creativity performance after the solo task. Females and mixed-gender couples in the not-joint condition significantly outperformed after the creativity task compared to males, who resulted as the weakest in all creativity dimensions after the not-joint condition.

These findings would be a preliminary evidence in favor of a *carry-over effect* from the motor task to the subsequent creativity task, which was modulated by gender composition. Therefore, in order to test this *carry-over effect*, we analyzed the relationship between tower-building time, gender composition and creativity performance in predictive terms. This analysis concerned only couples in the joint condition. Regression models showed that M–M couples’ execution time during the tower-building task predicted subsequent levels of creativity in terms of appropriateness, originality, and flexibility. In contrast, tower-building time did not predict creativity performance in F–F couples, and only marginally in mixed-gender couples (i.e., only for the creativity dimension of elaboration). Thus, results of regression were generally coherent with the observation that only M–M couples’ creativity performance benefited from the interpersonal movement coordination involved in the tower-building task, whereas F–F and cross-gender dyads showed superior creativity after the solo condition.

This research can provide some theoretical contributions to the field of creative collaboration in general and in particular to dyadic creativity. Prior studies on creativity have provided several evidences for understanding the individual or team-level determinants of successful collaboration (e.g., Andriopoulos, 2001; Sosa, 2011; Parjanen, 2012), but only little experimental research has been oriented to understand dyadic creativity and its determinants (e.g., Weinstein et al., 2010; Won et al., 2014). Specifically, although being in synch resulted as a key feature of highly creative dyads (e.g., Weinstein et al., 2010), no research has investigated the role of induced behavioral synchronization on a following creative performance. Therefore, the main aim of this study was to enhance our understanding of the determinants of interpersonal creativity by focusing, first, on interpersonal sensorimotor synchronization. To this end, we could not overlook the role of gender in shaping dyadic cooperative performance at the tower-building task. First, literature has evidenced that males tend to naturally coordinate better than females at a motor level (Thomas and French, 1985; Kauranen and Vanharanta, 1996; Hamill et al., 2005; Hall, 2006; Barrett et al., 2008; Chraif and Aniţei, 2013). We did not find the same pattern in our joint-action condition in which participants were required to cooperate (synchronize) to accomplish the same goal together (build a tower of cubes). Both males and females outperformed compared to mixed-gender couples: gender matters.

In line with this, we also observed that behavioral synchronization between partners was not only associated to dyadic creativity (Weinstein et al., 2010). Specifically, interpersonal synchronization fostered dyadic creativity, but only if considering also the role of dyads’ gender composition. This finding is not new to the wider literature on gender-diversity and group creativity (Campbell et al., 2013; Coursey et al., 2018). However, usually, heterogeneous groups showed better creative performance than homogeneous ones (e.g., McCroskey and McCain, 1974; Wood, 1987; Campbell et al., 2013). For instance, Schruijer and Mostert (1997) found that mixed-gender groups involved in creativity brainstorming tasks were more creative than same-gender ones, and other studies showed that couples of heterogeneous genders produced more ideas, made more associations among different ideas and took more viewpoints than same-gender ones (McCroskey and McCain, 1974; Wood, 1987). Here, we demonstrated that gender-diversity worked differently in dyads. Heterogenous couples showed the worst creative performance on each of the significant creativity dimensions (Quantity, Appropriateness, Flexibility). This is crucial since previous research on diversity and dyadic creativity have focused only on subjective variables (e.g., Cohen et al., 1960; Hoffman and Maier, 1961; Triandis et al., 1965; Torrance, 1970), while this is the first study that elucidated the role of gender, as a cross-domain “surface-level” variable (Horwitz and Horwitz, 2007, p. 990) on shaping dyadic interactions.

By including gender in our model, we moved forward also research in the sensorimotor synchronization domain. There is a common assumption on the role of interpersonal synchronization on other social or cognitive processes such as comprehension, cooperation, affiliation (Richardson and Dale, 2005; Hove and Risen, 2009; Wiltermuth and Heath, 2009; Valdesolo et al., 2010) suggesting that more (synchrony) means better (Abney et al., 2015; Paxton and Dale, 2017; Lozza et al., 2018). However, Abney et al. (2015) already evidenced that dyads’ gender composition combined with behavioral synchronization could influence the way in which interacting participants can develop productive forms of collaboration over time. Specifically, Cheng et al. (2015) speculated that increased interpersonal synchronization and high cooperative performance correlated in mixed-gender couples. However, the combined effect of gender and induced interpersonal synchronization on dyadic creativity was still an open question. This study brought evidence in favor of including gender in an effective model on dyadic creativity since it oriented the final creative outcomes differently. Only homogeneous pairs were more creative than mixed gender pairs after the tower-building task, which was designed to promote interpersonal synchronization. On the contrary, being in synch was detrimental for the creative performance of female and mixed-gender pairs. Surprisingly, interpersonal synchronization performance negatively influenced the ability to elaborate detailed creative ideas in mixed-gender couples. Being a member of a mixed-gender couple led to less detailed creative solutions. Despite diversity has been conceived as a source of new ideas and a facilitator of unusual combinations among them, some authors have suggested that it may be detrimental to interpersonal creativity since members need to coordinate in order to process new information and translate it into a concrete output (Huckman and Staats, 2011).

### Implications for Organizations

Since sensorimotor synchronization can be conceived as a basic cross-domain process, by testing its impact on dyadic creative performance, we could pave the way for designing new creativity-enhancing trainings, which can be potentially implementable into different domains, such as in the organizational field in which this topic is still emerging (e.g., Rouse, 2018). That is, if preliminary evidence exists regarding how to promote group creativity in organizational setting (Shalley et al., 2004; Anderson et al., 2014; Paulus et al., 2018), far less clear is how to promote dyadic creativity into organizations (e.g., Rouse, 2018).

Our results, albeit preliminary, could be useful to design novel strategies for improving creativity of pairs in organizational settings, i.e., by defining specific preparatory motor activities able to impact on dyadic creativity. For instance, some studies have evidenced that simple tasks such as tandem walking promoted a behavioral synchronization (Van Ulzen et al., 2008). A more engaging task to promote interpersonal synchronization could be the one developed by Fusaroli et al. (2016) using creative LEGO construction task. Also, the mirror game (in which participants are required to mimic each other alternatively) is a potential source of interpersonal synchronization (Hart et al., 2014; Cornejo et al., 2017). All these tasks could promote a sensorimotor flow of information between partners, which we postulated and demonstrated at the base of flow of ideas at the cognitive level. Crucially, this flow of information can be generated even between unacquainted partners, without prior arrangement or knowledge (Pezzulo et al., 2018). Therefore, these tasks could prime interpersonal scenarios typical in the organizational field, such as the meeting between a mentor with a new protégé. Mentoring practices are usually based on a dyadic relationship between a less experienced employee (i.e., the protégé) and a more expert individual (i.e., the mentor) aiming at integrating and including newcomers with diverse background within the same organization (Ragins and McFarlin, 1990). These practices, which are crucial for organizational advancement (Bolton and Humphreys, 1977) and involve an interplay of “diversities” (background, race, gender, culture), could be a fertile domain in which testing the effectiveness of this interpersonal synchronization task to foster dyadic creativity. With this regard, despite these mentoring practices have been recognized as crucial for general creativity retrospectively (Torrance, 1980, 1981, 1983, 1984, 2002), how to shape mentoring practices toward creativity outcomes is still an open issue (Form et al., 2017). With this regard, a future step could concern combining actual mentoring practices with an interpersonal synchronization training, maybe based on one of the above-mentioned synchronization tasks, in order to maximize the creative potential of the dyads composed by a mentor and a protégé.

### Limitations and Future Research

To our best knowledge, this is the first experiment showing a carry-over effect of a sensorimotor coordination task on subsequent dyadic creativity task. Due to the novel nature of the study, there are some limitations. First, a limitation of this work concerns the nature of the sample, which was small and composed of only students. However, our small sample size allowed for a mean power of 90% for interaction effects of target variables, that concerns our main hypotheses. Moreover, our aim was to study a cross-domain process (a form of interpersonal coordination, that is, behavioral synchronization), under controlled conditions. Therefore, future studies should consider also other populations and contexts, such as organizations, to test the extent to which our results are generalizable. Next, it should be noted that we selected a relatively “neutral” creativity task (i.e., brainstorming), which is usually adopted across different domains. Therefore, it could be useful to test whether our model holds even in domains of dyadic collaboration that are characterized by different levels of structuration of activities, such as within the design, engineering or artistic domains, or research collaboration.

Despite the preliminary nature of this study, results clearly evidenced that dyadic creativity can be also a function of gender and preparatory stages of a creativity task. Although our pattern of results slightly diverge from the body of studies on group creativity and diversity, it may be a preliminary experimental evidence that mechanisms underlying dyadic creativity are different from those concerning group creativity. With this regard, Rouse (2018) suggested that dyads may display different processes enabling collaborative creative work. For instance, creativity in dyads can don the guise of an intimate co-creation process in which ideas flows between partners sustained by a psychological pairing (Rouse, 2018) and constant feedback (Harrison and Rouse, 2014). Therefore, the degree of intimacy would make the difference with groups. Future studies should test these assumptions and consolidate, and further elucidate our findings.

Another useful future step could concern the role of other variables relevant for the organizational context, such as participants’ self-definition (Jung and Lee, 2015) or coping styles in relation to gender (Watson et al., 2011). Specifically, it would be useful to study creativity dynamics in organizations starting from a leader-employee unit of analysis, which is emerging as a promising field of analysis (e.g., Mittal and Dhar, 2015). This would be far more crucial if our synchronization task was able to trigger specific collective efficacy-related process (Bandura, 1977, 2000), which resulted as key factors in the relationship between managers and employees (e.g., Chen and Bliese, 2002; George, 2007; Paulus and Kenworthy, 2017), as well as drivers of team creativity (Kim and Shin, 2015) and group’ general performance (Zaccaro et al., 1995). With this regard, in this study, males’ natural better performance at motor coordination task could have promoted higher sense of collective efficacy stemming from the behavioral performance itself and turned into higher creativity levels. Finally, collective efficacy resulted also sensitive to gender differences (Lee and Farh, 2004).

However, literature on collective efficacy has focused on general group performance (not on creativity) without considering a previous task. Therefore, we could only assume that, in this study, females could have felt more competent when they performed the task alone (in this study, we refer to the “solo condition”), and this could have acted a driver of higher collective efficacy, which was translated into better dyadic creative performance. Finally, in mixed-couples the natural males’ ability to coordinate could have been dampened by the females’ behavioral contribution to the task, thus decreasing collective efficacy. In short, it would be useful to elucidate whether collective efficacy is related to gender diversity and dyadic creativity on the base of the task in which the dyads had been previously involved.

## Conclusion

Despite organizations are populated with countless dyadic relationships, how to unlock the creative potential of these links is still an open issue. The current work adds new insight into how dyadic creativity can be shaped by a combination of a previous synchronization task and gender. By introducing synchronization-conductive tasks, it would be possible to boost creative performance in a differentiated way, thus potentially impacting on organizational effectiveness.

## Author Contributions

AC and AnG conceived the main idea of the article. EF collected all data. FF, PC, EF, and AC carried out statistical analyses. AC and AnG wrote the first draft, while AlG, EF, SG, AD, PC, and GR contributed to the final writing and editing of the manuscript. AC and AnG supervised the entire work. All authors read and approved the final version and contributed according to their competences and interests.

## Conflict of Interest Statement

The authors declare that the research was conducted in the absence of any commercial or financial relationships that could be construed as a potential conflict of interest.
